# Case report: a Chinese girl like atypical Rubinstein–Taybi syndrome caused by a novel heterozygous mutation of the *EP300* gene

**DOI:** 10.1186/s12920-022-01424-4

**Published:** 2023-02-16

**Authors:** Zhouxian Bai, Gaopan Li, Xiangdong Kong

**Affiliations:** 1 The Genetics and Prenatal Diagnosis Center, The Department of Obstetrics and Gynecology, The First Affiliated Hospital of Zhengzhou University, Zhengzhou, 450052 Henan China; 2 The Department of Pediatrics, The First Affiliated Hospital of Zhengzhou University, Zhengzhou, 450052 Henan China

**Keywords:** *EP300*, Novel mutation, Atypical Rubinstein–Taybi syndrome, Immunodeficiency

## Abstract

**Background:**

Rubinstein–Taybi syndrome (RSTS) is an extremely rare autosomal dominant inheritable disorder caused by *CREBBP* and *EP300* mutations, while atypical RSTS harbouring variant from the same genes but not obvious resembling RSTS. There are only a few cases of Menke–Hennekam syndrome (MKHK) with variant of exon 30 or 31 of *CREBBP* or *EP300* gene have been reported that not resembling RSTS recent years. Atypical RSTS cannot be accurately classified as MKHK, nor is it easy to identify the obvious classic characteristics of RSTS. The clinical manifestations and genetic variation of atypical RSTS are not fully understood.

**Case presentation:**

We present a Chinese core family with a girl had recurrent respiratory tract infection and developmental delay. The patient with language and motor mild development retardation, she has slight abnormal facial features, mild hirsutism and post-axial hexadactylia of left foot. Her cisterna magna is enlarged to connect with the fourth ventricle, and the ventricular system is enlarged. She has a malacia beside the posterior horn of the left lateral ventricle. The patient has primary low immunoglobulin G and A, but her level of immunoglobulin M content in blood is normal. The patient harbors a novel heterozygous frameshift variant of c.2499dupG in exon 14 of *EP300* gene, that it is proved to de novo origin. The mutation is judged to be a pathogenic mutation, and it has high-grade pathogenic evidence.

**Conclusion:**

The clinical and genetic evaluation of this case corroborates that clinical features caused by c.2499dupG in exon 14 of *EP300* are less marked than RSTS2 patient although it is difficult to establish an accurate genotype–phenotype correlation. Our additional case also helps to deepen the clinical and genetic spectrum in this disorder. The case provides a novel mutation of *EP300* and enriches the phenotypes related with the gene. We have contributed new variation and disease information for guardians and doctors to broaden the knowledge about *EP300*-RSTS genotype and phenotype, this may contribute to ameliorate the health management of patients and improve the genetic counseling to the families.

**Supplementary Information:**

The online version contains supplementary material available at 10.1186/s12920-022-01424-4.

## Background

Rubinstein–Taybi syndrome (RSTS; OMIM #180849, #613684) is a known disorder characterized by a distinctive face, broad thumbs, broad halluces and big toes, short stature, and variable degree of intellectual disability [1, 2]. It is a rare autosomal dominant genetic pluri-malformative syndrome, which well-defined facial features include down slanting palpebral fissures, convex nasal bridge, columella below alae nasi, and a characteristic grimacing smile [3]. The other aspects of manifestation include growth retardation, microcephaly and behavioural problems [3]. Broad and angulated thumbs and halluces are usually considered as hallmarks for clinical diagnosis [4]. RSTS1 (OMIM #180849) is caused by variants of *CREBBP* that coding CREB-binding protein acted as transcriptional co-activators, and RSTS2 (OMIM #613684) is caused by variants of its paralog *EP300* that coding E1A associated protein p300. *CREBBP* and *EP300* mutations have been identified in majority (50–60%) and minority (3–5%) of RSTS affected individuals, respectively [4–6], most, if not all, of them occur de novo.

Recently Menke–Hennekam syndrome (MKHK, OMIM #618332, #618333) is put forward as an entity caused by the same gene *CREBBP* and *EP300* mutations but not resembling Rubinstein–Taybi syndrome [7, 8]. The manifestations of MKHK include variable impairment of intellectual development and specific facial appearance, but patients with MKHK do not resemble the striking phenotype of RSTS. The other symptoms of MKHK are also consist of feeding difficulties, autistic behavior, recurrent upper airway infections, hearing impairment, and short stature, microcephaly are also frequently seen. Current evidence suggests that MKHK2 (OMIM #618333) is caused by heterozygous mutations in exon 30 or 31 of the *EP300* gene, which symptom is milder than MKHK1 (OMIM #618332) caused by heterozygous mutations in exon 30 or 31 of the *CREBBP* gene [9, 10]. Here we report one further child with one frameshift variant, although outside exon 30 or 31 of *EP300*, without the typical RSTS features.

## Case presentation

A Chinese family from central China was recruited for this study. The patient was a girl at 4 years of age, she was hospitalized for recurrent respiratory infections and pneumonia in the past half 1 year. The sufferer's parents are 33 years old and healthy. The patient sought medical and genetic consultations in the hospital from September 2020 to February 2021. Written informed consent has been signed for the collection and use of clinical materials. In this research, we chose a family-based strategy to determine the exact inheritance pattern and candidate disease causing gene mutation. Using such a family-based strategy, we can also determine whether phenotype and genotype co-segregate in the family, which helps to estimate the pathogenicity of candidate mutation and explore the relationship between phenotype and genotype. The proband was screened by whole exome sequencing (WES), then, the parents were tested by Sanger sequencing to detect and verify the carrying status of candidate mutation screened through WES.

The patient is the first child of nonconsanguineous Chinese parents born at 41 weeks gestation, following an unremarkable pregnancy with a birth weight of 3.1 kg. At birth, her left foot was noted to be hexadactylia. She was low neonatal response. Her parents and family feel that she has a peculiar appearance, neither like father nor mother. There are no concerns about her hearing and vision, but eye-tracking and listen-tracking was poor during infancy. From 3 to 8 months, she received a 6-month rehabilitation training for movement development retardation. She rolled over at aged 7 months, sat unsupported at aged 9 months, crawled at aged 12 months and walked independently at aged 16 months. At 2 years of age, she received rehabilitation training for language delay. She is mild growth retardation. She had no self-injurious behavior. She often pushes other children, but is sociable and interactive. At present, she is height of 101 cm, weight of 15.5 kg and head circumference of 49 cm, which are at low level among the normal range (103.1 ± 3.9 cm, 16.17 ± 1.73 kg, 49.4 ± 1.3 cm) of girls aged 4 years. She can ride a tricycle independently and freely (Additional file 1: Short Video S1). She also builds blocks and draws with no problem (Additional file 1: Short Videos S2 and S3). Her language comprehension is somewhat poor. She can now express long sentences or recite simple enlightening poems of the Tang dynasty such as *singing goose*, *spring dawn* and so on. But when she said long sentences, she sometimes would miss one word. She is now in kindergarten middle shift.

**Fig. 1:**
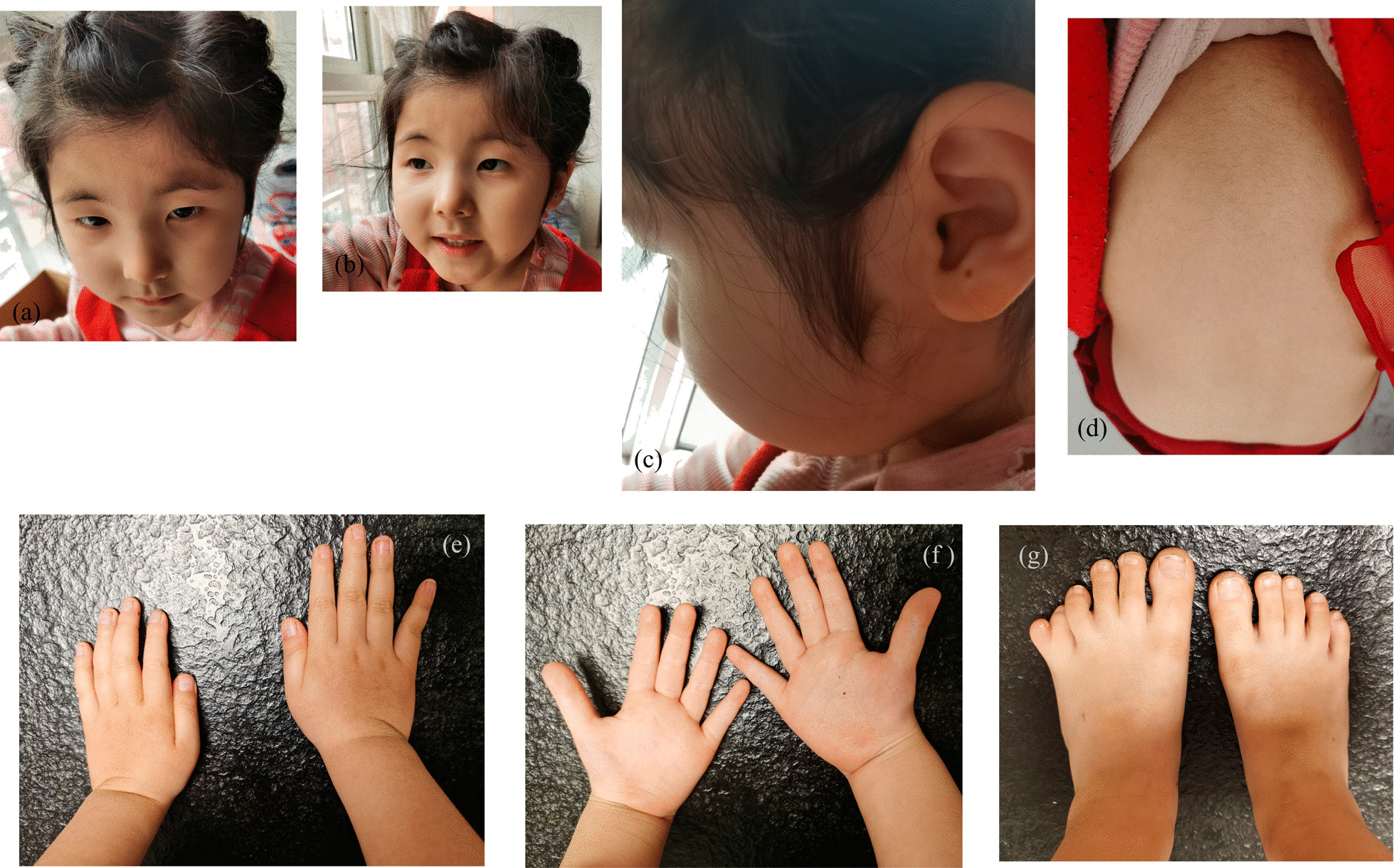
Phenotypic features of patient described in this study. **a** and **b** The patient 4 years old. Note slightly arched eyebrows and synophridia, a square tip to his nose, normal columella, prominent two front teeth, normal tooth number and absence of characteristic grimace of Rubinstein–Taybi syndrome. **c** The fine hairs on the front of the ear and on the cheek are hair whorl. **d** and **e** The child has heavy fine hair on her back and opisthenar. **f** Patient has no broad or angulated thumbs, nor broad distal phalanges of the fingers, as seen in patients with Rubinstein–Taybi syndrome. **g** Girl has a sixth toe of her left foot, that hexadactyly

Patient’s facial and physical features are shown in Fig. 1 and Additional file 1: Fig. S1a–g. She at 4 years of age, has slightly arched eyebrows, synophridia, long eyelashes, a square tip to his nose, normal columella, prominent two front teeth and normal tooth number. She is absence of microcephaly, beaked nose and characteristic grimacing smile like RSTS. The fine hairs on the front of the ear and on the cheek form a hair whorl. The child has hirsute back. She has no broad or angulated thumbs, nor broad distal phalanges of the fingers, as seen in patients with RSTS. Her brain plain scan of MRI shows as follows. Punctate long T1 and long T2 signal shadow was seen beside the posterior horn of left lateral ventricle; FLAIR showed high signal outside and low signal inside; DWI showed low signal with high b value, and the lesion had no space occupying effect. It indicated that softening lesion of the posterior horn of left lateral ventricle. The enlarged cisterna magna was connected with the fourth ventricle, the ventricular system was enlarged, and there was no widening and deepening phenomenon in the cerebral sulcus, cisterna and cerebral fissure. The morphology, structure and signal intensity of cerebellum and brainstem were normal. The midline structure did not deviate.

The patient has been hospitalized for 5 times due to recurrent respiratory infections with or without pneumonia in the last half year. The patient was found to have primary low immunity, transient EB virus infection and splenomegaly. Her examination results of immune globulins content in peripheral blood or urine are presented in Table 1. It indicates that she suffers primary low immunoglobulin G/A. She has normal level of immunoglobulin M.

**Table 1:** Content of immune globulins in peripheral blood or urine of the patient

IG Type	T1	T3	T4	T5*	T6*	T7*	RRB	T2	RRU (mg/mL)
IgG	4.77↓	3.71↓	3.62↓	21.68	8.22	7.42	5.4–13.4 mg/L	4.67↓	5.9–14.3
IgA	0.14↓	0.12↓	0.09↓	0.31	0.15↓	0.10↓	0.3–1.88 g/L	0.17↓	0.38–2.22
IgM	1.03	1.39	1.37	1.42	1.54	1.43	0.43–1.93 g/L	1.42	0.45–2.08

*IG* immune globulin, *RRB* reference range in blood, *RRU* reference range in urine

^*^Denotes after intravenous immunoglobulin G therapy. T1, 2020.08.28; T2, 2020.10.24; T3, 2020.11.22; T4, 2020.12.10; T5, 2020.12.27; T6, 2021.01.25; T7, 2021.02.28

**Fig. 2:**
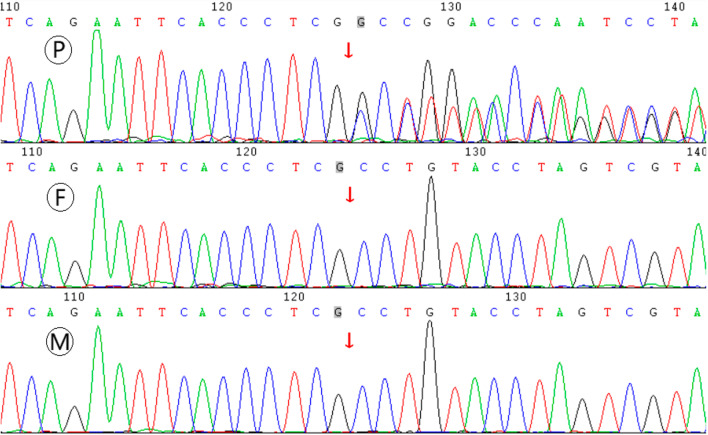
Sanger sequencing results of *EP300* c.2499dupG mutation in the core family. Sample P denotes the patient, sample F and M denote the father and mother of the patient, respectively

Candidate disease causing mutation detected in the patient through WES, and it was verified by Sanger sequencing. The parents were also tested by Sanger sequencing to detect the carrying status of candidate mutation (Fig. 2). The patient harbors a heterozygous mutation of c.2499dupG in exon 14 of *EP300* gene, which encodes the adenovirus E1A-associated cellular p300 transcriptional co-activator protein. NM_001429 was chosen as transcript reference sequence of *EP300* gene during NGS data analysis. The genetic relationship between parents and daughter was confirmed by paternity test. Subsequent Sanger sequencing tests displayed that none of the parents carried the *EP300* c.2499dupG mutation, and it proved to be de novo origin.

**Fig. 3:**
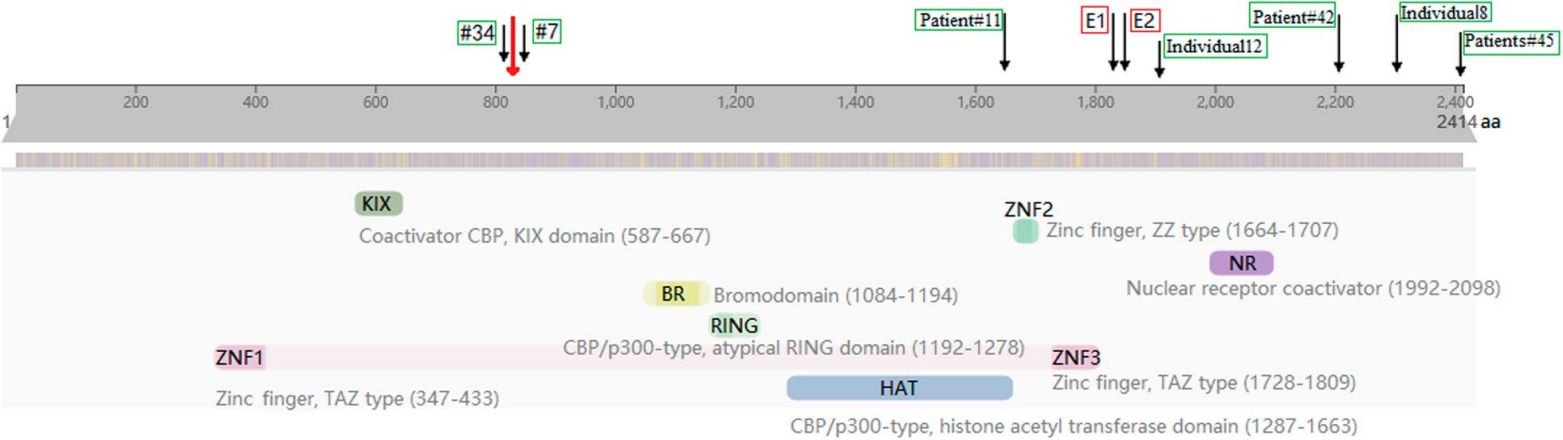
Distribution of *EP300* domains and mutations in our patient (in red arrow) versus in previous patient cohort (in black arrow). Schematic representation of the *EP300* protein and functional domains excerpted from http://www.ebi.ac.uk/interpro/ at March 26th 2021. Previous patient cohort refers to the cohort published [7, 14–16]. Previous patients marked by red box accorded with the diagnosis of MKHK, the other previous patients marked by green box accorded with the diagnosis of RSTS

*EP300* c.2499dupG is a frameshift mutation in exon 14, which is presumed to cause amino acid sequence changes of p.Pro834Alafs*4. It results in substitution of amino acid 834th and termination of translation after four codons shift, which is sited in a region, not domain, of the protein with still uncharacterized function (Fig. 3). The c.2499dupG p.(Pro834Alafs*4) variant in our patient is a novel that not been related to disease recorded in HGMD database. The variant is not found in all populations or the East Asian population in gnomAD database, so minor allele frequency of the rare mutation is unknown (extremely low). Due to nonsense mediated decay of frameshift mutations, truncated nonfunctional product proteins may be generated, and such mutation will be deleterious. According to ACMG guidelines, the novel frameshift mutation of *EP300* c.2499dupG p.(Pro834Alafs*4) should be considered pathogenic due to its high evidence grade of pathogenicity (PVS1 + PS2 + PM2).

## Discussion

Mutations in *CREBBP* and *EP300* caused RSTS has been well-known, while the patients with missense variant in exon 30/31 of *CREBBP* and *EP300* didn't have typical characteristics of RSTS that reported in recently publications [7, 8, 10]. *CREBBP* is originally isolated as a nuclear protein with intrinsic histone or non-histone acetyltransferase activity, which is ubiquitously expressed and involved in the transcriptional co-activation of a variety of transcription factors through binding to cAMP-response element binding protein (CREB) [11]. The protein of *CREBBP* also functions as a scaffold to stabilize additional protein interactions with the transcription complex [12]. *EP300* encodes the adenovirus E1A-associated cellular p300 transcriptional co-activator protein that is significant in the processes of cell proliferation and differentiation. Its product protein acts as histone acetyltransferase participating in chromatin remodeling to regulate the process of transcription. And *EP300* also mediates cAMP-gene regulation through specifically binding to phosphorylated CREB protein [12]. There is very high sequence similarity in homologous area of bromo-domain, cysteine-histidine-rich regions, and histone acetyltransferase domain of the proteins of *EP300* and *CREBBP*. They play important roles in embryonic development, growth control, and homeostasis, which implements through coupling chromatin remodeling to transcription factor recognition.

There are 25 patients reported in the medical literature without the typical RSTS features and with exon 30/31 missense or in-frame deletion *CREBBP* variants so far [7, 8, 10]. The main features of MKHK1 caused by *CREBBP* mutations include developmental delay, distinctive facial features, autistic behavior, feeding difficulties, recurrent upper airway infections, and brain abnormalities revealed by MRI. An additional Chinese case with one *CREBBP* in-frame deletion variant in the beginning of exon 30 has atypical RSTS phenotypes [13]. Now it seems that she is consistent with the diagnosis of MKHK1, although the mutation in the HAT domain where no pathogenic variants have been previously reported to be responsible for MKHK. The 2-year-7-month-old Chinese girl presented facial dysmorphism of MKHK included telecanthus, a depressed nasal ridge, short nose, anteverted nares, short columella, and long philtrum. Her other symptoms were mild cognitive impairments, developmental delay, short stature, recurrent upper airway infections, and mild thinning of corpus callosum revealed by MRI.

Some scholars call the atypical RSTS phenotypes with *EP300* variant Menke–Hennekam syndrome 2 [7]. The main typical characteristics of previous patient E1 and E2 regarded as MKHK2 include variable impairment of intellectual development and facial dysmorphisms, and feeding difficulties, autistic behavior, recurrent infections are also frequently seen. Combined with the analysis of clinical manifestations of previous MKHK1 cases caused by *CREBBP* exon 30/31 mutations, Menke–Hennekam syndrome is described as a congenital disorder characterized by variable impairment of intellectual development and facial dysmorphisms that do not resemble the striking phenotype of RSTS [7, 8], and MKHK2 is milder than MKHK1. Patient E1 with *EP300* c.5471A > C p.(Gln1824Pro) had recurrent otitis, airway infections and urinary tract infections owing to low immunoglobulins, and was diagnosed with autism spectrum disorder. She necessitated a percutaneous gastrostomy (PEG) when as a neonate. Patient E2 with *EP300* c.5492_5494del p.(Arg1831del) continued to have recurrent otitis, and autism spectrum disorder was also diagnosed (Fig. 3). Her language and motor delayed. Brain MRI was not performed in these patients.

The number of MKHK2 cases caused by *EP300* gene mutation is still small. The previously reported patients [14] with an *EP300* variant in exon 31 could reconsider the name of the disease according to the new concept. Individual 8 with *EP300* c.6915_6918del p.(Asn2305Lysfs*47) was male, his striking phenotypes included mild specific facial appearance, varying degrees of speech and motor retardation, reactive airway disease, undescended testicle and severely restricted growth, without broad thumb and radial deviation of the hand. Individual 8 was mild RSTS2, but cannot be attributed to MKHK2. His demise caused by severe cardiac involvement, which may be a few and isolated phenotype. While Individual 12 with *EP300* frameshift variant of c.5720delC p.(Pro1907Leufs*53) had classical characteristics of RSTS. These two patients also did not do brain MRI. There were five persons with *EP300* exon 30/31 variants previously reported as RSTS2 patients (Fig. 3) [15]. The Patient #42 was a 9 years old boy with an *EP300* in-frame deletion variant of c.6627_6638del p.(Asn2209_Gln2213delinsLys) in exon 31, but he had the typical manifestations of RSTS. The Patient #11 was a 18 years old male youth with an *EP300* frameshift variant of c.4954_4957dup p.(Cys1653Tyrfs*21) in exon 30, and both of the Patients #45 of mother and daughter had *EP300* frameshift variant of c.7222_7223del p.(Gln2408Glufs*39) in exon 31. They were all RSTS2 patients with variable disease symptoms, respectively. Patients #45 have milder clinical manifestation than Patient #11, and even Patients #45 with the same mutation in one same family also realized the heterogeneity of clinical features of the disease.

We also compare our case with the other 2 cases harbouring a nonsense variant of the same exon 14 reported in Fergelot's paper [16]. The case #7 harbours c.2554C > T (p.Gln852*) heterozygous variation and the case #34 harbours c.2437C > T (p.Gln813*) heterozygous variation. Since there are no facial photos of these two male patients, we mainly refer to the descriptive information in the literature. These two cases of #7 and #34 have more typical facial RSTS characteristics than our case to some extent, such as microcephaly, columella below alae nasi and low-set ears, etc. They have more noticeable specific facial appearance, but no hypertrichosis. The most significant difference between our case and the #7 and #34 cases is that histories of recurrent respiratory infections and primary low immunity, the two previous cases have no recurrent respiratory infections and immune problems.

These above investigations have expanded our knowledge about the spectrum of diverse phenotypes driven by dissimilar molecular and cellular consequences resulting from different class of variants in the same gene [10]. However, by comparison, we can see that it is not exactly the same as Menke and Hennekam proposed that ‘Menke–Hennekam syndrome is caused by missense mutations in the last part of exon 30 and beginning of 31 of the CREBBP/*EP300* gene resulted in a gain of function. Mutations elsewhere in the gene causing Rubinstein–Taybi syndrome, result in haplo-insufficiency or perturb the function of a domain, specifically the HAT’. The previous Chinese case with, paralog of *EP300*, one *CREBBP* in-frame deletion variant in the 5′ end of HAT domain of beginning exon 30 has phenotypes more similar to typical MKHK rather than RSTS, and while *EP300* in-frame deletion variant in exon 31 not always cause MKHK2 (previous Patient #42). Disease caused by mutations outside the exon 30/31 of *EP300* sometimes is not always typical Rubinstein–Taybi syndrome [3, 14, 15].

Our patient has slightly arched eyebrows and synophridia, mild hirsutism, post-axial hexadactylia of left foot, and with no beaked nose or other striking features of RSTS. She has mild development retardation of language and motor, abnormality of brain structure was seen by MRI. The patient had a history of multiple respiratory tract infections and primary low immunity. All these clinical manifestations suggest that our patient is consistent with the phenotype of atypical RSTS, and she harbours a de novo frameshift *EP300* variant of c.2499dupG p.(Pro834Alafs*4) in exon 14 that not involved HAT domain. *EP300* related RSTS phenotype is often less classical than that related to pathogenic *CREBBP* variants, so it would be more difficult to differentiate between RSTS and MKHK. On the other hand, taking into account that only two patients with *EP300* variants and MKHK have been officially described, it is appropriate for this Chinese girl to be diagnosed as atypical RSTS. Looking back at these patients with *EP300* mutations, we found that a history of recurrent infection or even primary low immunity is a notable feature of the disorder. Some scholars believe that variants causing RSTS in other parts of *CREBBP/EP300* would result in haplo-insufficiency of functions or perturb the function of the HAT domain, typically [7]. But not all functions of *CREBBP/EP300* are dose-dependent. The disease caused by *EP300* mutation shows phenotype heterogeneity and likely constitutes at least two different entities, such as RSTS and MKHK. Due to the relatively small number of patients with *EP300* mutations reported, there are still a lot of details about these two entities that need to be constructed. This case contributes one novel de novo *EP300* variant and novel phenotypes to the atypical RSTS. It further extends the borders of the *EP300* variant resulting in RSTS-like disease and expands its clinical spectrum.

## Conclusion

This case report demonstrates that clinical features with c.2499dupG p.(Pro834Alafs*4) in exon 14 of *EP300* are less marked than in RSTS2 patient, although previous studies have suggested that mutations beyond the exons 30 and 31 of *EP300* gene can lead to RSTS. Our additional case could help to deepen the clinical and genetic spectrum in this disorder, so as to establish the correlationship between phenotypes and genotypes to some extent. Our case provides a novel *EP300* mutation for atypical RSTS and enriches the phenotypes. This could contribute to improve the health management and genetic counselling for patients, and facilitate clinical research in a certain extent.

## Supplementary Information

**Additional file 1: Fig. S1a–g.** Phenotypic features of patient described in this study. **a** and **b** The patient 4 years old. Note slightly arched eyebrows and synophridia, a square tip to his nose, normal columella, prominent two front teeth, normal tooth number and absence of characteristic grimace of Rubinstein–Taybi syndrome. **c** The fine hairs on the front of the ear and on the cheek are hair whorl. **d** and **e** The child has heavy fine hair on her back and opisthenar. **f** Patient has no broad or angulated thumbs, nor broad distal phalanges of the fingers, as seen in patients with Rubinstein–Taybi syndrome. **g** Girl has a sixth toe of her left foot, that hexadactyly. **Short Video S1.** She can ride a tricycle independently and freely. **Short Video S2 and S3.** She can build blocks and draw with no problem.

## Data Availability

The raw datasets used and analyzed during the current study are not deposited in publicly available repositories because of considerations about the security of human genetic resources. Any questions should be directed to the corresponding author. We provide conclusive variant information without identifying/confidential patient data in the paper or its appendix. For other details of the availability of data and material, please refer to the methods section of the article.

## Abbreviations

NGS: Next-generation sequencing
gnomAD: Genome aggregation database
HGMD: The human gene mutation database at the institute of medical genetics in Cardiff
ACMG: The American College of Medical Genetics and Genomics
MRI: Magnetic resonance imaging
FLAIR: Fluid attenuated inversion recovery
DWI: Diffusion weighted imaging
