# Supplementary figures and images for: Case report: a Chinese girl like atypical Rubinstein–Taybi syndrome caused by a novel heterozygous mutation of the EP300 gene
Source: BMC Med Genomics. 2023 Feb 16;16:24. doi: 10.1186/s12920-022-01424-4 (PMC9933371; doi:10.1186/s12920-022-01424-4)

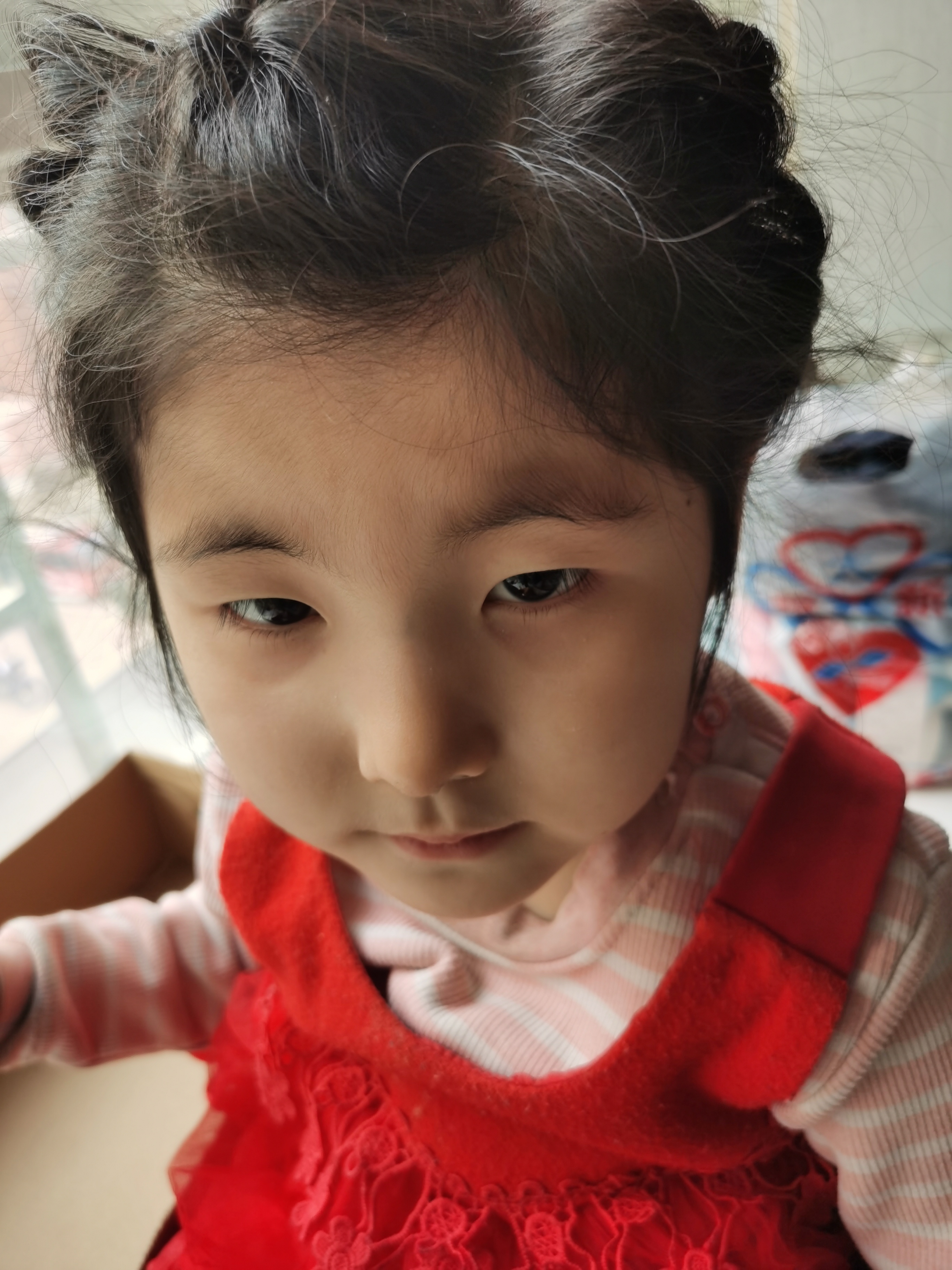

Supplement: Supplementary file 1 — Additional file 1: Fig. S1a–g. Phenotypic features of patient described in this study. a and b The patient 4 years old. Note slightly arched eyebrows and synophridia, a square tip to his nose, normal columella, prominent two front teeth, normal tooth number and absence of characteristic grimace of Rubinstein–Taybi syndrome. c The fine hairs on the front of the ear and on the cheek are hair whorl. d and e The child has heavy fine hair on her back and opisthenar. f Patient has no broad or angulated thumbs, nor broad distal phalanges of the fingers, as seen in patients with Rubinstein–Taybi syndrome. g Girl has a sixth toe of her left foot, that hexadactyly. Short Video S1. She can ride a tricycle independently and freely. Short Video S2 and S3. She can build blocks and draw with no problem. [file 12920_2022_1424_MOESM1_ESM.zip › 12920_2022_1424_MOESM1_ESM/Figure S1a.jpg]

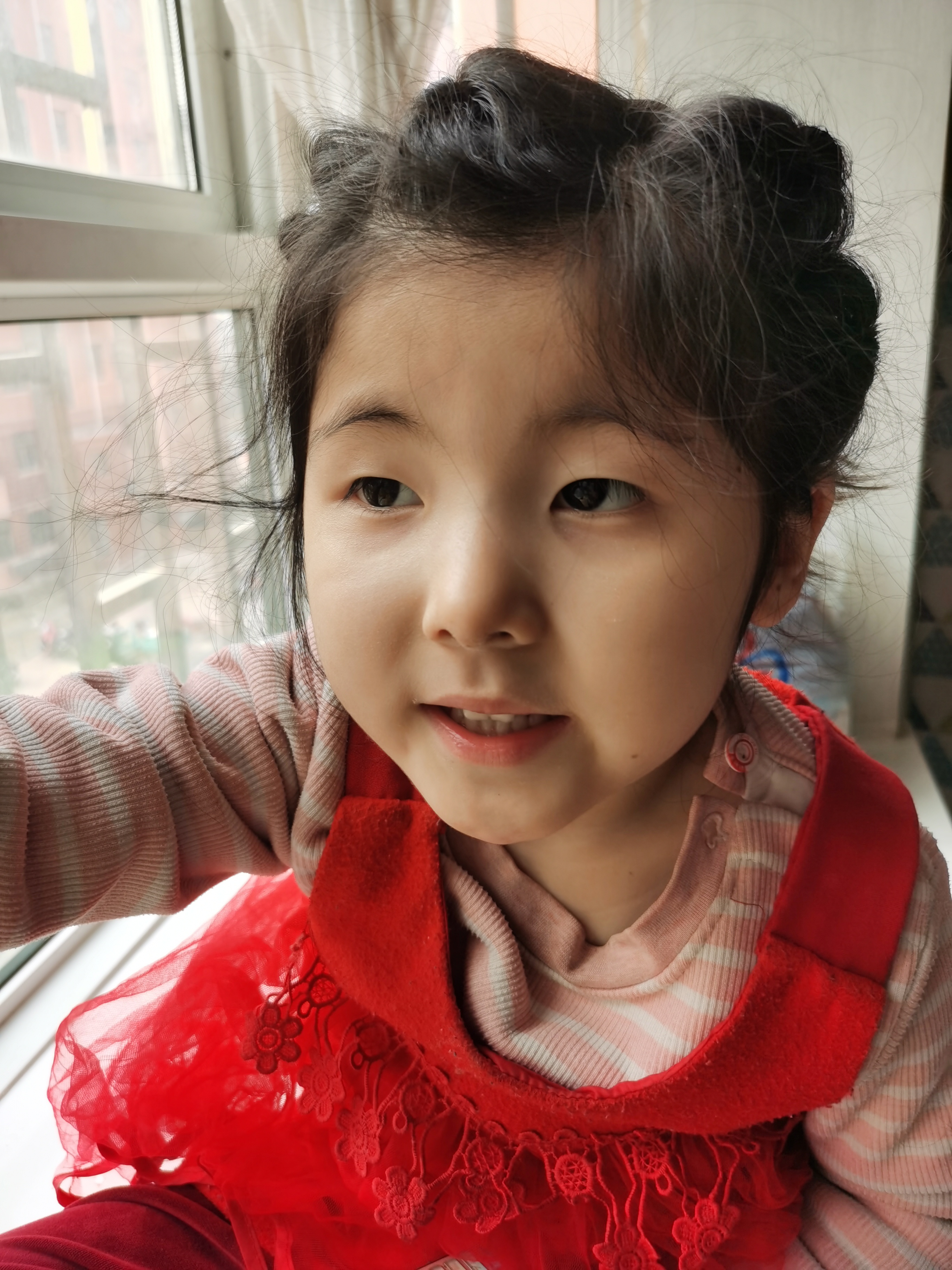

Supplement: Supplementary file 1 — Additional file 1: Fig. S1a–g. Phenotypic features of patient described in this study. a and b The patient 4 years old. Note slightly arched eyebrows and synophridia, a square tip to his nose, normal columella, prominent two front teeth, normal tooth number and absence of characteristic grimace of Rubinstein–Taybi syndrome. c The fine hairs on the front of the ear and on the cheek are hair whorl. d and e The child has heavy fine hair on her back and opisthenar. f Patient has no broad or angulated thumbs, nor broad distal phalanges of the fingers, as seen in patients with Rubinstein–Taybi syndrome. g Girl has a sixth toe of her left foot, that hexadactyly. Short Video S1. She can ride a tricycle independently and freely. Short Video S2 and S3. She can build blocks and draw with no problem. [file 12920_2022_1424_MOESM1_ESM.zip › 12920_2022_1424_MOESM1_ESM/Figure S1b.jpg]

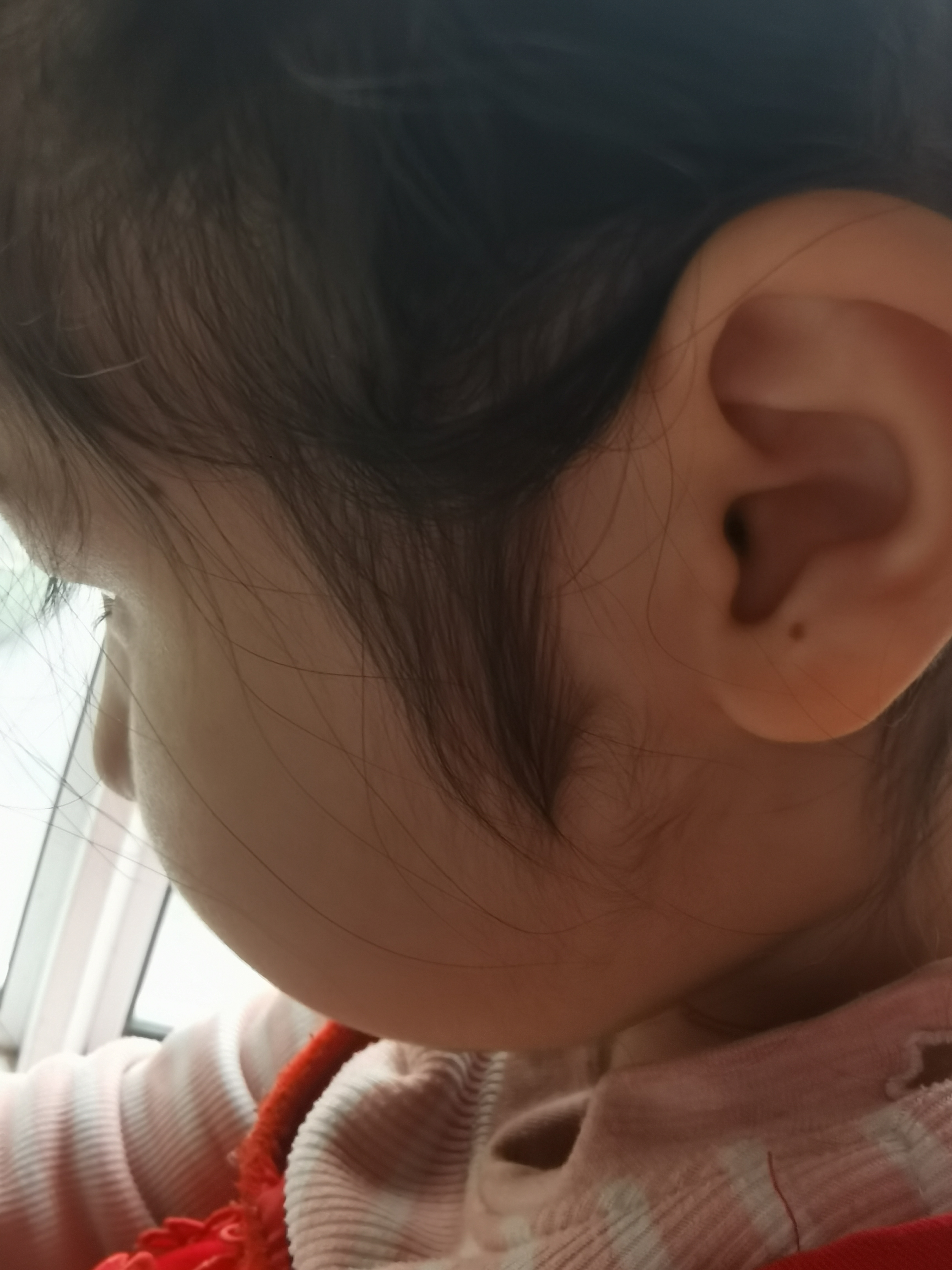

Supplement: Supplementary file 1 — Additional file 1: Fig. S1a–g. Phenotypic features of patient described in this study. a and b The patient 4 years old. Note slightly arched eyebrows and synophridia, a square tip to his nose, normal columella, prominent two front teeth, normal tooth number and absence of characteristic grimace of Rubinstein–Taybi syndrome. c The fine hairs on the front of the ear and on the cheek are hair whorl. d and e The child has heavy fine hair on her back and opisthenar. f Patient has no broad or angulated thumbs, nor broad distal phalanges of the fingers, as seen in patients with Rubinstein–Taybi syndrome. g Girl has a sixth toe of her left foot, that hexadactyly. Short Video S1. She can ride a tricycle independently and freely. Short Video S2 and S3. She can build blocks and draw with no problem. [file 12920_2022_1424_MOESM1_ESM.zip › 12920_2022_1424_MOESM1_ESM/Figure S1c.jpg]

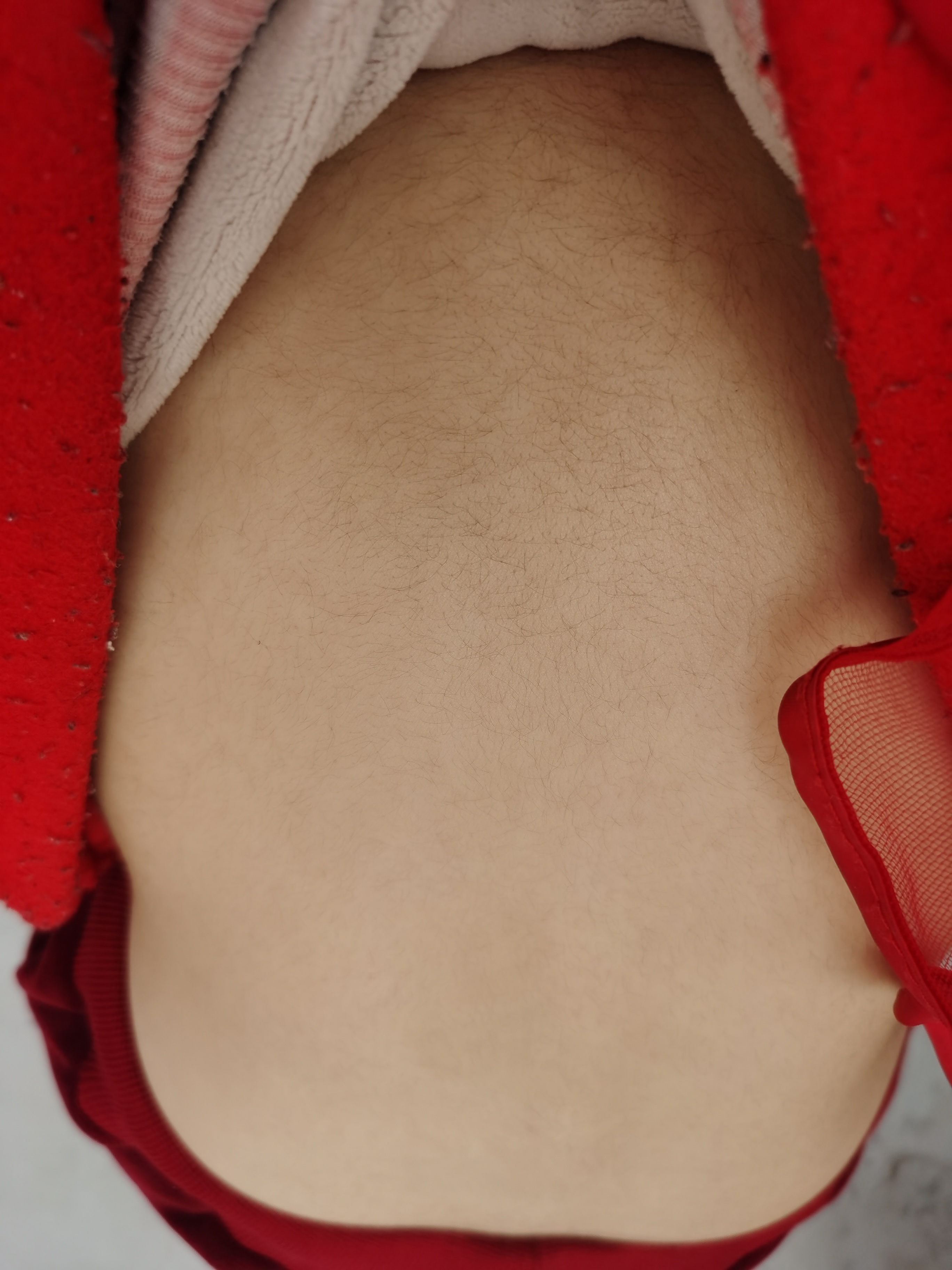

Supplement: Supplementary file 1 — Additional file 1: Fig. S1a–g. Phenotypic features of patient described in this study. a and b The patient 4 years old. Note slightly arched eyebrows and synophridia, a square tip to his nose, normal columella, prominent two front teeth, normal tooth number and absence of characteristic grimace of Rubinstein–Taybi syndrome. c The fine hairs on the front of the ear and on the cheek are hair whorl. d and e The child has heavy fine hair on her back and opisthenar. f Patient has no broad or angulated thumbs, nor broad distal phalanges of the fingers, as seen in patients with Rubinstein–Taybi syndrome. g Girl has a sixth toe of her left foot, that hexadactyly. Short Video S1. She can ride a tricycle independently and freely. Short Video S2 and S3. She can build blocks and draw with no problem. [file 12920_2022_1424_MOESM1_ESM.zip › 12920_2022_1424_MOESM1_ESM/Figure S1d.jpg]

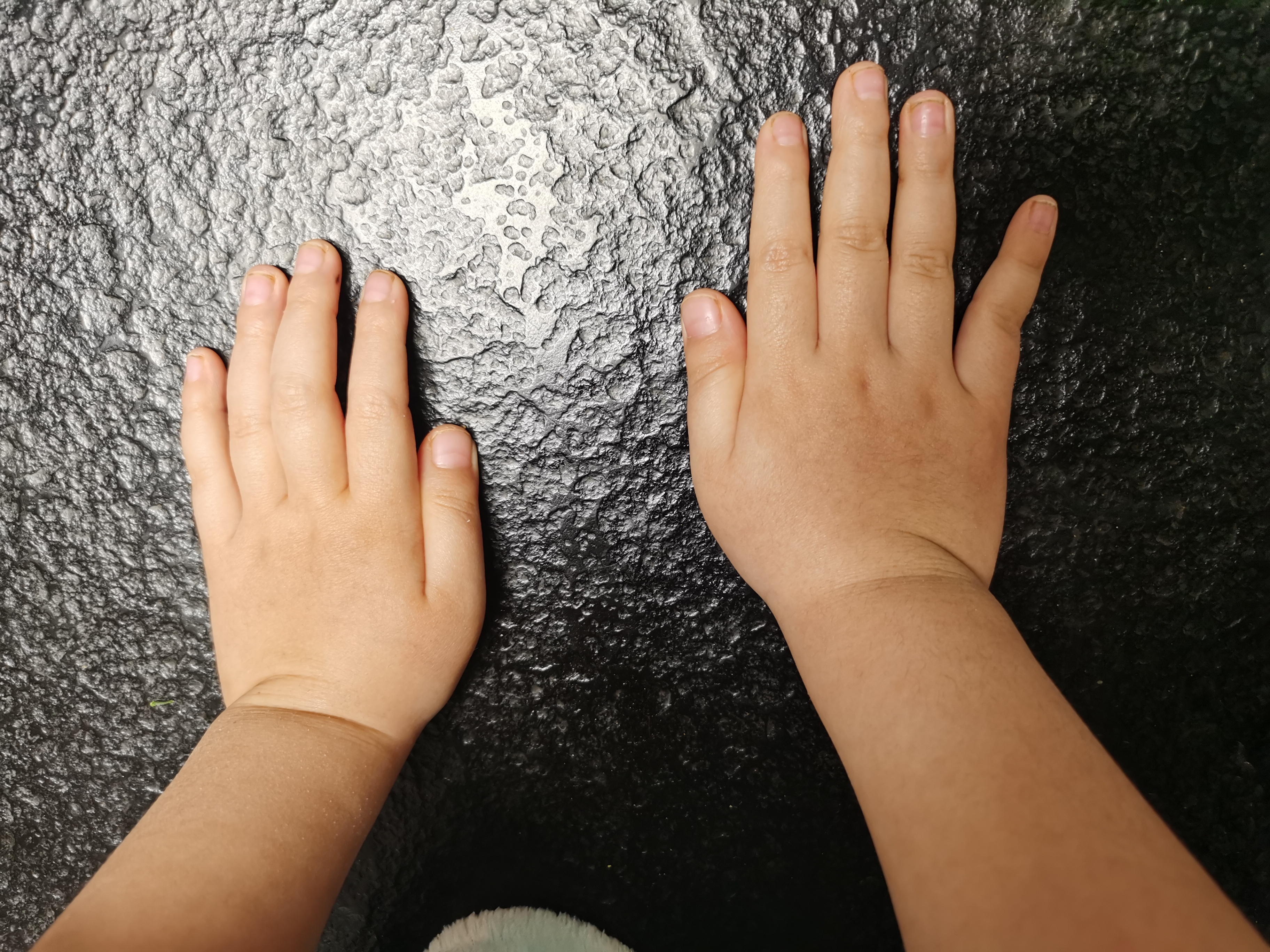

Supplement: Supplementary file 1 — Additional file 1: Fig. S1a–g. Phenotypic features of patient described in this study. a and b The patient 4 years old. Note slightly arched eyebrows and synophridia, a square tip to his nose, normal columella, prominent two front teeth, normal tooth number and absence of characteristic grimace of Rubinstein–Taybi syndrome. c The fine hairs on the front of the ear and on the cheek are hair whorl. d and e The child has heavy fine hair on her back and opisthenar. f Patient has no broad or angulated thumbs, nor broad distal phalanges of the fingers, as seen in patients with Rubinstein–Taybi syndrome. g Girl has a sixth toe of her left foot, that hexadactyly. Short Video S1. She can ride a tricycle independently and freely. Short Video S2 and S3. She can build blocks and draw with no problem. [file 12920_2022_1424_MOESM1_ESM.zip › 12920_2022_1424_MOESM1_ESM/Figure S1e.jpg]

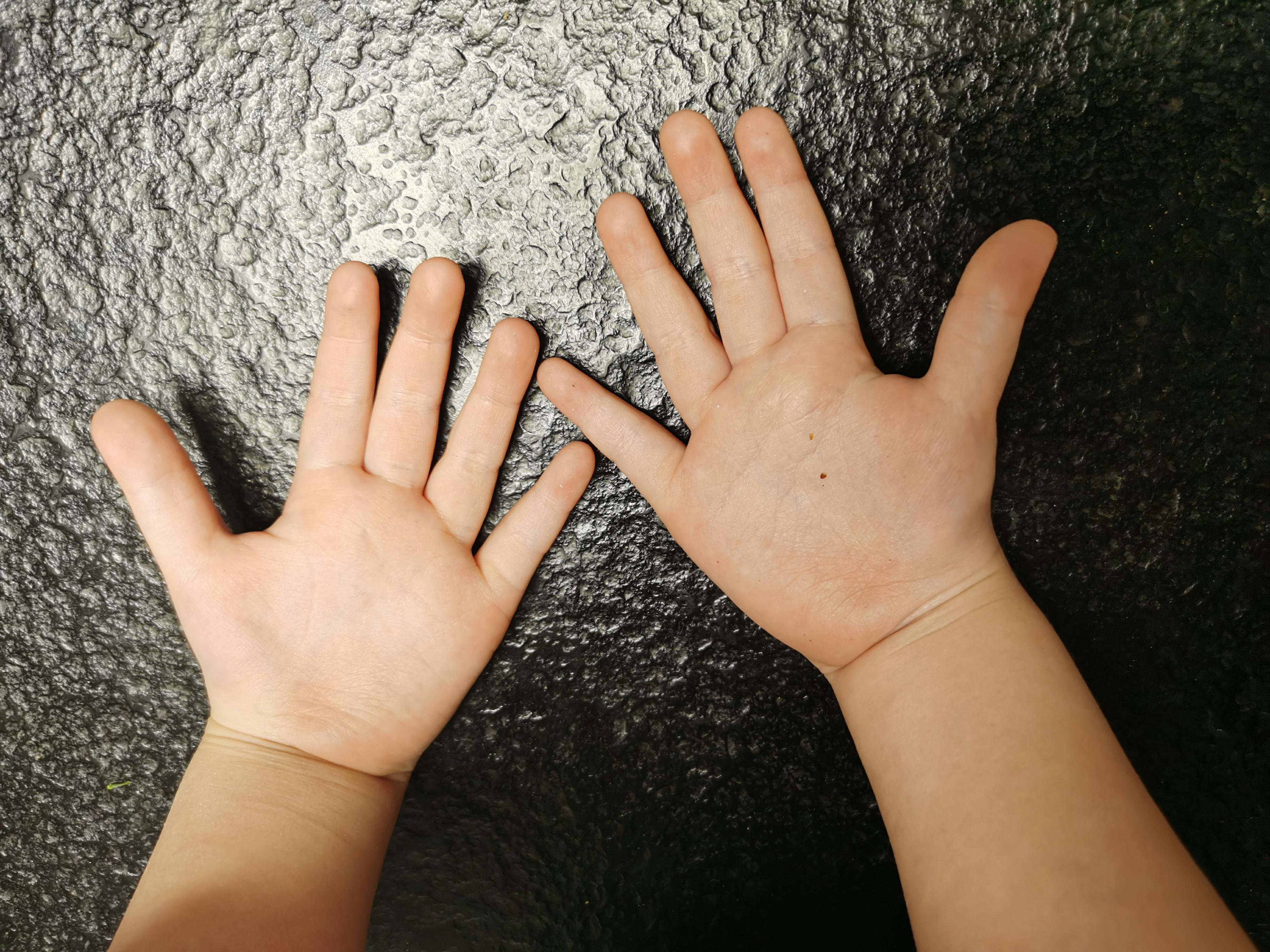

Supplement: Supplementary file 1 — Additional file 1: Fig. S1a–g. Phenotypic features of patient described in this study. a and b The patient 4 years old. Note slightly arched eyebrows and synophridia, a square tip to his nose, normal columella, prominent two front teeth, normal tooth number and absence of characteristic grimace of Rubinstein–Taybi syndrome. c The fine hairs on the front of the ear and on the cheek are hair whorl. d and e The child has heavy fine hair on her back and opisthenar. f Patient has no broad or angulated thumbs, nor broad distal phalanges of the fingers, as seen in patients with Rubinstein–Taybi syndrome. g Girl has a sixth toe of her left foot, that hexadactyly. Short Video S1. She can ride a tricycle independently and freely. Short Video S2 and S3. She can build blocks and draw with no problem. [file 12920_2022_1424_MOESM1_ESM.zip › 12920_2022_1424_MOESM1_ESM/Figure S1f.jpg]

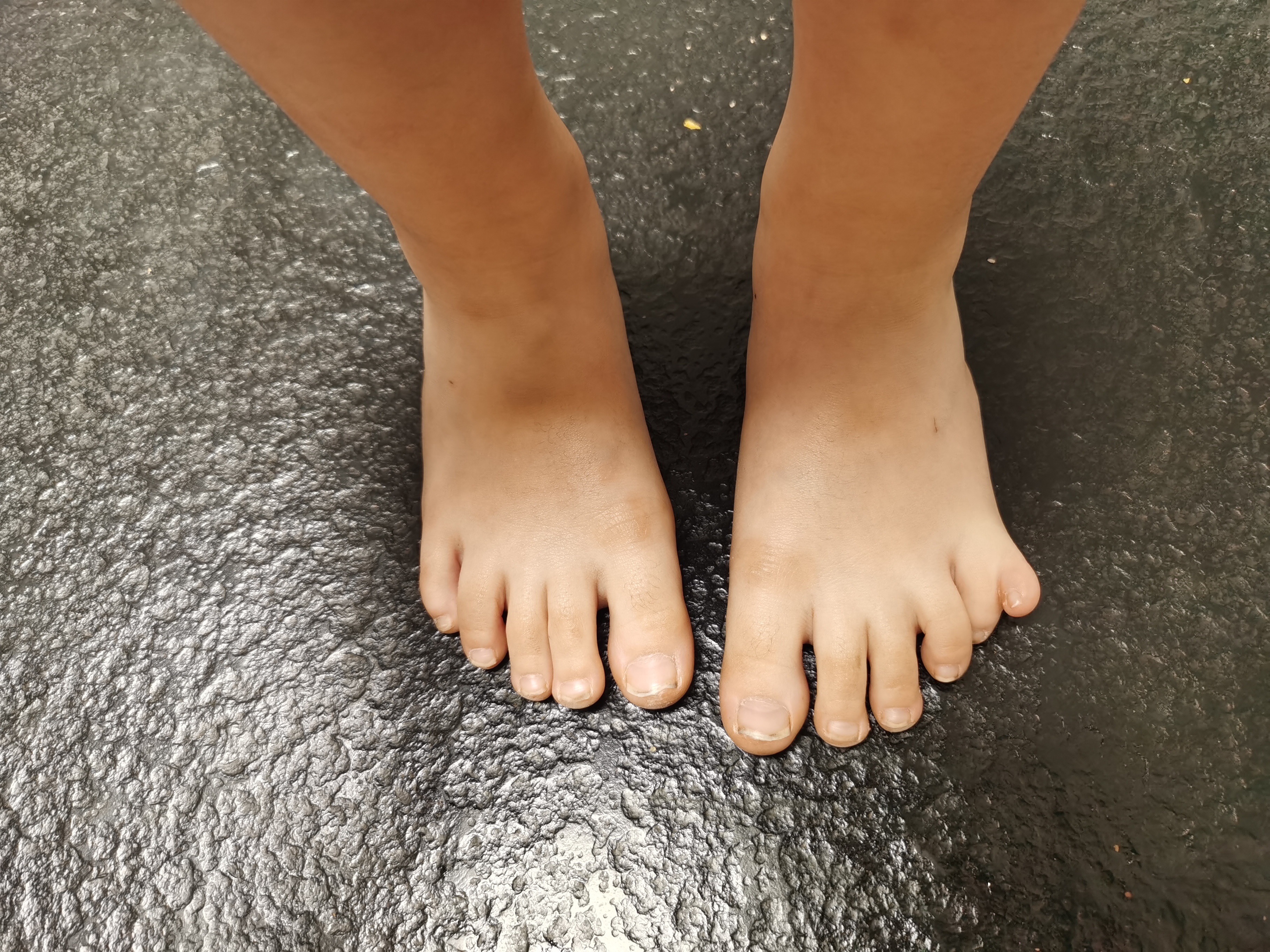

Supplement: Supplementary file 1 — Additional file 1: Fig. S1a–g. Phenotypic features of patient described in this study. a and b The patient 4 years old. Note slightly arched eyebrows and synophridia, a square tip to his nose, normal columella, prominent two front teeth, normal tooth number and absence of characteristic grimace of Rubinstein–Taybi syndrome. c The fine hairs on the front of the ear and on the cheek are hair whorl. d and e The child has heavy fine hair on her back and opisthenar. f Patient has no broad or angulated thumbs, nor broad distal phalanges of the fingers, as seen in patients with Rubinstein–Taybi syndrome. g Girl has a sixth toe of her left foot, that hexadactyly. Short Video S1. She can ride a tricycle independently and freely. Short Video S2 and S3. She can build blocks and draw with no problem. [file 12920_2022_1424_MOESM1_ESM.zip › 12920_2022_1424_MOESM1_ESM/Figure S1g.jpg]
